# Associations of glycemic status with dynamic disease trajectories of atrial fibrillation and dementia

**DOI:** 10.1016/j.tjpad.2024.100047

**Published:** 2025-01-13

**Authors:** Chenglong Li, Daijun He, Yufan Liu, Chao Yang, Luxia Zhang, Rodica Pop-Busui

**Affiliations:** a National Institute of Health Data Science at Peking University, Beijing 100191, PR China; b Institute of Medical Technology, Health Science Center of Peking University, Beijing 100191, PR China; c Renal Division, Department of Medicine, Peking University First Hospital, 8 Xishiku Street, Xicheng District, Beijing 100034, PR China; d Institute of Nephrology, Key Laboratory of Renal Disease, Ministry of Health of China; and Key Laboratory of Chronic Kidney Disease Prevention and Treatment (Peking University), Ministry of Education, Beijing, PR China; e Research Units of Diagnosis and Treatment of Immune-mediated Kidney Diseases, Chinese Academy of Medical Sciences, Beijing, PR China; f Capital Medical University, Beijing, PR China; g Advanced Institute of Information Technology, Peking University, Hangzhou 311215, PR China; h State Key Laboratory of Vascular Homeostasis and Remodeling, Peking University, Beijing 100191, PR China; i Division of Metabolism, Endocrinology and Diabetes, Department of Internal Medicine, University of Michigan, Ann Arbor, MI, USA

**Keywords:** Glycemic status, Atrial fibrillation, Dementia, Disease trajectory, Comorbidity

## Abstract

**Background:**

Atrial fibrillation (AF) has been associated with elevated dementia risk, while few studies have examined the role of the optimal glycemic status in disease trajectories of AF and dementia.

**Objectives:**

We aim to evaluate associations between glycemic status with disease trajectories of AF and dementia, as well as major dementia subtypes, including Alzheimer's disease and vascular dementia.

**Design:**

Population-based cohort study.

**Setting:**

UK Biobank.

**Participants:**

A total of 458 368 participants who were free of prevalent dementia and AF at baseline, with complete glycemic status assessment.

**Measurements:**

Based on clinical recommendations, we categorized glycemic status as low-normal (glycated hemoglobin [HbA1c] <5.5 %), normal (HbA1c 5.5 to 5.9 %), pre-diabetes (HbA1c 6.0 to 6.4 %), diabetes with HbA1c<7 %, and diabetes with HbA1c≥7 %. Outcomes including AF, dementia (all-cause and sub-type dementia), and death were ascertained via linkage to external registry databases. A multi-state survival analysis was conducted to evaluate disease trajectories of AF and dementia.

**Results:**

Better glycemic status was consistently associated with decreased hazards of trajectories of AF and dementia, including progression from AF to the comorbidity of AF and dementia. Among people with diabetes, those with HbA1c<7 % had a 31 % lower hazard (hazard ratio [HR], 0.69; 95 % confidence intervals [CI], 0.51–0.93) of progression from incident AF to dementia comorbidity, compared to those with HbA1c≥7 %. Similar risk reductions were found in individuals with pre-diabetes, normal HbA1c, and low-normal HbA1c, respectively. Strong dose-response associations were observed, with each 1 % increment in HbA1c related to a 28 % higher hazard of progression from AF to dementia comorbidity (HR,1.28; 95 % CI, 1.19–1.37). The glycemic status was most relevant for associations with disease trajectories of AF and vascular dementia, compared to trajectories of AF and Alzheimer's disease.

**Conclusions:**

The better glycemic status was consistently associated with lower hazards of disease trajectories of AF and dementia, including the reduced risk of progression from incident AF to comorbidity of AF and dementia. These findings support the significance of reaching optimal glycemic status to alleviate the huge disease burden of both AF and dementia simultaneously.

## 1 Introduction

Atrial fibrillation (AF) remains the most prevalent type of cardiac rhythm abnormalities, with rising disease burden [1]. The disability-adjusted life years due to AF has increased from 3.79 million in 1990 to 8.39 million in 2019, while the number of prevalent cases has nearly doubled to 59.7 million since 1990 [1]. Incident AF has also been associated with elevated risks of cardiovascular events and mortality [2]. Moreover, as most AF cases remain undetected in practice, the related health threats could be significantly underestimated [3], highlighting the importance of primary prevention at the population level.

Dementia has become a non-neglectable public health challenge, accounting for substantial proportions of mortality and disability around the world [4]. Previous studies have observed AF was strongly associated with elevated dementia incidence, with potential mechanisms including hypoperfusion, inflammation, atherosclerotic vascular disease, microhemorrhage, and brain atrophy [[5], [6], [7], [8]]. Our previous contribution also linked an earlier AF onset age with higher dementia risk [9]. Nevertheless, previous contributions have investigated AF and dementia onset in an isolated fashion, without accounting for the disease trajectories, e.g. the development courses of the two diseases with time. Moreover, few investigations were conducted to identify modifiable risk factors contributing to the disease trajectories of AF and dementia.

Among identified modifiable risk factors, hyperglycemia and diabetes have been consistently associated with both AF and dementia [4,10]. It has been well-established that diabetes precipitates AF occurrence and contributes to worsen prognosis of AF, such as elevated risks of stroke, AF recurrence, and cardiovascular mortality [10]. Nevertheless, prospective studies on associations between glycemic status assessed using glycated hemoglobin (HbA1c) and AF onset are scarce. Some reports showed that a strict glycemic control, defined as HbA1c <7 % (53 mmol/mol) by the American Diabetes Association (ADA) [11], was consistently associated with a lower dementia incidence in individuals with diagnosed diabetes [12,13]. However, few studies have assessed disease trajectories of AF and dementia. Additionally, no prospective studies have examined associations between glycemic status with disease trajectories of AF and dementia, leaving important knowledge gaps impeding efforts targeting primary prevention and prognosis improvement.

Therefore, the current study aimed to investigate the associations between glycemic status with disease trajectories of AF and dementia, including trajectories of AF and all-cause dementia and its major subtypes. We also aimed to evaluate the exposure-response associations between the HbA1c level with trajectories of AF and dementia.

## 2 Methods

### 2.1 Study population

Approved by the North West Multi-centre Research Ethics Committee, the UK Biobank is established with the aim to lay foundations for comprehensively investigating risk factors of major chronic diseases. More than 500 000 men and women aged 40–69 years from 22 assessment centers in England, Scotland, and Wales were recruited during 2006–2010, with informed consent obtained. Further details regarding the design and the survey content are described elsewhere [14]. This study followed the Strengthening the Reporting of Observational Studies in Epidemiology (STROBE) reporting guideline.

Among the original 502 369 participants available for inclusion, we excluded 228 participants with prevalent dementia, 35 970 participants lacking glycemic status assessments, and 7803 participants with prevalent AF, leaving 458 368 participants for final analysis. The detailed sample selection procedure is presented in **Supplementary Figure 1**.

### 2.2 Glycemic status

Irrespective of diabetes diagnosis, HbA1c was measured in a central laboratory for all participants at recruitment. Diabetes diagnosis was ascertained by combining self-reported or doctor-diagnosed diabetes diagnosis, reported usage of anti-hyperglycemic medications or insulin, or the measured HbA1c >6.5 % (48 mmol/mol) [15]. Based on standard clinical cut-off points [16], we categorized glycemic status as low-normal (HbA1c<35 mmol/mol or <5.5 %), normal (HbA1c 35 to 41 mmol/mol or 5.5 to 5.9 %), pre-diabetes (HbA1c 42 to 47 mmol/mol or 6.0 to 6.4 %), diabetes with HbA1c<7 % (53 mmol/mol), and diabetes with HbA1c≥7 % (53 mmol/mol), as embraced by previous studies [11,17]. We also used the ADA recommended cut-off points of pre-diabetes of HbA1c 5.7–6.4 % for sensitivity analysis. The HbA1c level on the continuous scale was also analyzed (per 1 % increment).

### 2.3 Outcome ascertainment

We ascertained AF diagnosis via three approaches: 1) the International Classification of Diseases (ICD)−10 codes, obtained via linkage to hospital admissions records and death certificate records [18]; 2) the Office of Population Censuses and Surveys' Classification of Surgical Operations version-4 (OPCS-4), which is a statistical classification for clinical coding of hospital interventions and procedures undertaken by the NHS; 3) self-reported diagnosis of clinical conditions (for prevalent AF diagnosis only). Further details regarding the ascertainment procedure and codes are presented in **Supplementary Table 1**.

Incident dementia cases in UK Biobank were assessed using a validated algorithm, incorporating records of hospital admissions and death registry. The ICD-9 and ICD-10 codes were used to identify all-cause and sub-type dementia cases, with details presented in **Supplementary Table 2**. Sub-type dementia cases included Alzheimer's disease and vascular dementia. The performance of the algorithm has been externally validated, with positive predictive values of 84.5 % for all-cause dementia, 70.8 % for Alzheimer's disease, and 33.3 % for vascular dementia [19]. We defined the comorbidity of AF and dementia as diagnoses with AF or dementia at first and further the other. Follow-up years were calculated from baseline until the date of events of interest (AF, dementia, and comorbidity) or death or loss to follow-up, or December 31, 2022, whichever came first.

### 2.4 Covariates

Based on previous studies, we selected covariates for adjustment, including demographics (age, sex, and ethnicity), socioeconomic factors (education attainment, employment status, and family income), lifestyle behaviors (alcohol consumption, moderate or vigorous physical activity participation, and current smoking), and prevalent major chronic diseases (hypertension, chronic kidney disease, and cardiovascular diseases other than AF) [10,12,20]. A touchscreen questionnaire was used to collect information on demographics, socioeconomic factors, and behaviors. Prevalent chronic diseases were ascertained by combining self-reported diagnosis, medication use, laboratory measurements, and linkage to registry data (death register and hospital inpatient admission records).

### 2.5 Statistical analysis

For descriptive statistics, the mean (standard deviation [SD]) or median (inter-quartile range [IQR]) was used for continuous variables, and numbers and percentages for categorical variables. Differences between groups were tested using analysis of variance, chi-square test, or Kruskal-Wallis test.

To comprehensively evaluate the associations between glycemic status and disease trajectories of AF and dementia, we conducted a multi-state survival analysis using the multi-state Markov model. The multi-state Markov model is an extension of the traditional Cox proportional hazards model, capable of handling multiple competing events as states of disease trajectories and assessing the associations of risk factors with different stages of disease progression simultaneously. The model has been well-embraced for assessing disease trajectories in epidemiological studies [21,22]. We considered five states when building the multi-state model, including baseline (free of AF and dementia), incident AF, incident dementia, comorbidity of AF and dementia, and all-cause mortality. Accordingly, eight trajectories were predefined: 1) baseline to AF; 2) baseline to dementia; 3) baseline to death; 4) incident AF to comorbidity; 5) incident AF to death; 6) incident dementia to comorbidity; 7) incident dementia to death; 8) comorbidity to death. For participants with identical recorded dates of disease and death, a time-interval of 0.5 days was introduced according to a previous study [21]. Age was used as the time scale for the trajectory analysis, as identical to the previous study [21]. Adjusted hazard ratios (HR) and 95 % confidence intervals (CI) were estimated to reflect associations between glycemic status with hazards of disease trajectories of AF and dementia. In conjunction with the multi-state model, we assessed the exposure-response relationships between HbA1c (per 1 % scale) with hazards of specific trajectories between AF and dementia. Restricted cubic spline functions were used to depict the non-linear dose-response association curve. According to model fitting statistics (Akaike information criterion and Bayesian information criterion), four knots were selected for curve smoothing. Widely used locations of knots were applied, fixed at the 5th, 35th, 65th, and 95th percentiles of exposure variables [23].

Several sensitivity analyses were conducted. First, in addition to the all-cause dementia, we further accounted for disease trajectories of incident AF and dementia subtypes, including trajectories of incident AF, Alzheimer's disease, and vascular dementia, respectively. Dose-response relationships between HbA1c level with hazards of the above trajectories were also evaluated. Second, given the potential impact of prevalent cardiovascular conditions on both glycemic status, AF, and dementia risk, we excluded individuals with prevalent cardiovascular diseases, including stroke, heart failure, and coronary heart disease. Third, we re-categorized glycemic status according to the ADA criteria, by changing the cut-off points of pre-diabetes to HbA1c of 5.7 to 6.4 %, and repeated primary analysis. Fourth, we further controlled for levels of blood glucose and blood pressure (both systolic and diastolic blood pressure), included as continuous variables. Fifth, as some participants were diagnosed of AF and dementia at the same date, we modified the pre-defined disease trajectories, by further including the transition from baseline to the comorbidity of AF and dementia. Then we repeated the multi-state analysis. Sixth, mediation effect of incident AF was explored in associations between glycemic status and dementia risk. Finally, to evaluate selection bias, a non-response analysis was conducted comparing baseline characteristics of included and excluded participants.

Statistical analysis was conducted using SAS 9.4 (SAS Institute, Cary, NC) and R language 4.3.1 (R Foundation, Vienna, Austria), with a two-tailed alpha of 0.05 considered statistically significant.

## 3 Results

As shown in Table 1, among 458 368 participants included for analysis (mean [SD] age, 56.4 [8.1] years, 45.4 % men), participants with better glycemic status were younger, more likely to be women and of white ethnicity, had better socioeconomic status, higher adherence to regular physical activity, and less prevalent chronic diseases (all *P* < 0.001, Table 1). The median (IQR) HbA1c was 5.2 % (5.0–5.4) for participants of low-normal HbA1c (*n* = 295 154), 5.7 % (5.6–5.8) for participants of normal HbA1c (*n* = 120 794), 6.1 % (6.0–6.2) for participants of pre-diabetes (*n* = 14 201), 6.3 % (5.8–6.6) for participants of diabetes with HbA1c<7 % (*n* = 17 152), and 7.8 % (7.3–8.7) for participants of diabetes with HbA1c≥7 % (*n* = 11 067), respectively (*P* < 0.001 for comparison, Table 1).

**Table 1:** Baseline characteristics of participants.

Characteristics	Low-normal HbA1c N = 295 154	Normal HbA1c N = 120 794	Pre-diabetes N = 14 201	Diabetes with HbA1c<7 % N = 17 152	Diabetes with HbA1c≥7 % N = 11 067	P*
Age, mean (SD), years	55.1 (8.2)	58.7 (7.3)	60.0 (6.9)	59.5 (7.3)	58.7 (7.3)	<0.001
Men	131 650 (44.6)	52 748 (43.7)	6640 (46.8)	10 033 (58.5)	6881 (62.2)	<0.001
White ethnicity	283 710 (96.1)	112 368 (93.0)	12 144 (85.5)	14 937 (87.1)	9460 (85.5)	<0.001
Higher education	146 605 (49.7)	52 505 (43.5)	5415 (38.1)	6457 (37.6)	4139 (37.4)	<0.001
Annual income ≥ £31 000	146 547 (49.7)	45 174 (37.4)	4178 (29.4)	5068 (29.5)	3171 (28.7)	<0.001
Employed	272 765 (92.4)	111 041 (91.9)	12 785 (90.0)	14 815 (86.4)	9352 (84.5)	<0.001
Current smoking	26 171 (8.9)	16 657 (13.8)	2375 (16.7)	1947 (11.4)	1316 (11.9)	<0.001
Alcohol intake once per week	215 545 (73.0)	79 061 (65.5)	7918 (55.8)	9506 (55.4)	5480 (49.5)	<0.001
Physical activity ≥ 150 min/week	217 339 (73.6)	85 005 (70.4)	9071 (63.9)	10 606 (61.8)	6476 (58.5)	<0.001
Chronic kidney disease	5335 (1.8)	3795 (3.1)	695 (4.9)	1254 (7.3)	1052 (9.5)	<0.001
Hypertension	146 709 (49.7)	74 053 (61.3)	10 527 (74.1)	13 962 (81.4)	9196 (83.1)	<0.001
Cardiovascular disease	12 145 (4.1)	9846 (8.2)	2062 (14.5)	3284 (19.1)	2165 (19.6)	<0.001
HbA1c, median (IQR), %	5.2 (5.0–5.4)	5.7 (5.6–5.8)	6.1 (6.0–6.2)	6.3 (5.8–6.6)	7.8 (7.3–8.7)	<0.001

Abbreviations: HbA1c, glycated hemoglobin.

⁎ Group differences tested using analysis of variance, chi-square test, or Kruskal-Wallis test.

### 3.1 Disease trajectories of af and dementia

**Fig. 1:**
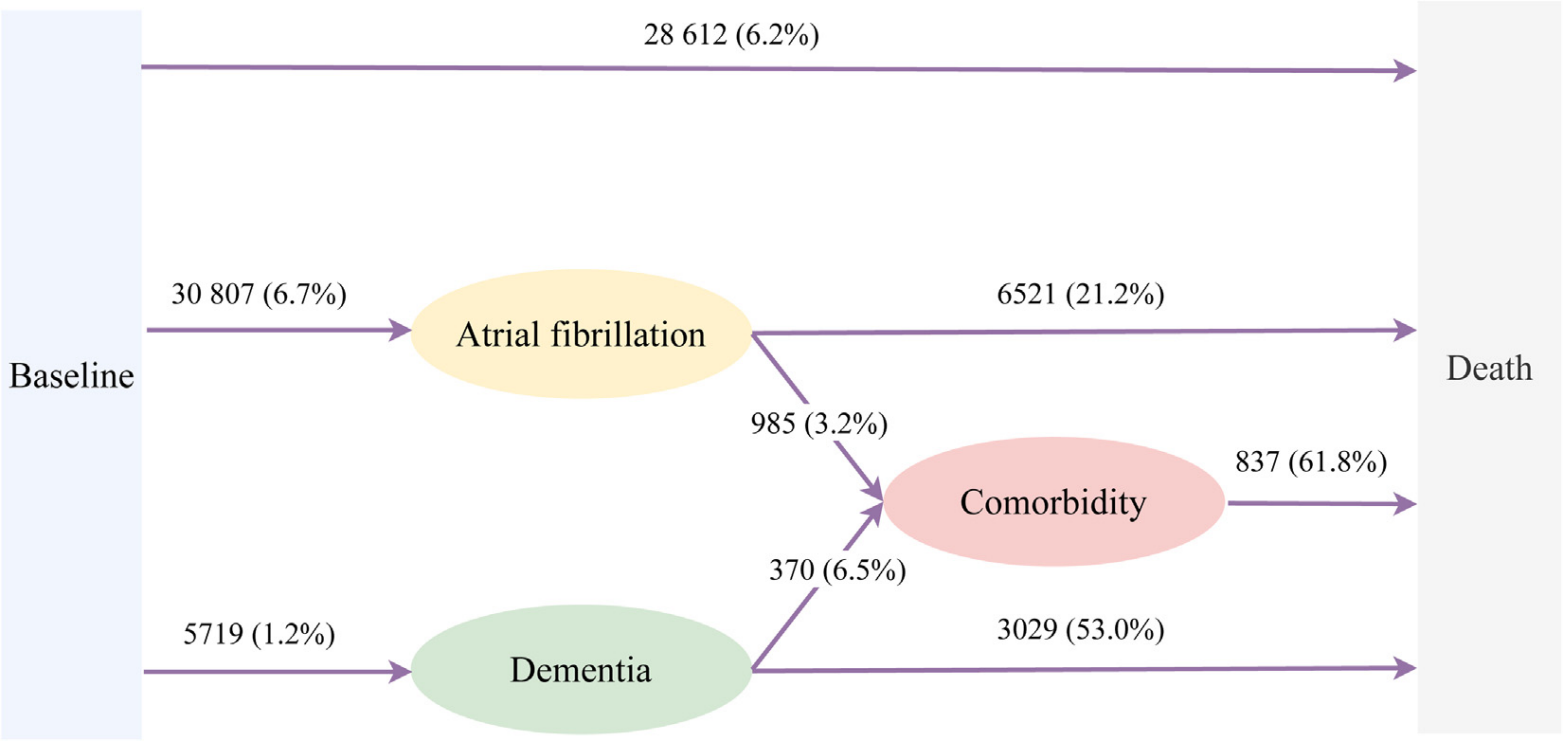
Disease trajectories of atrial fibrillation and dementia. Observed trajectories (expressed as numbers and percentages of participants in the previous stage) of atrial fibrillation and dementia.

**Fig. 2:**
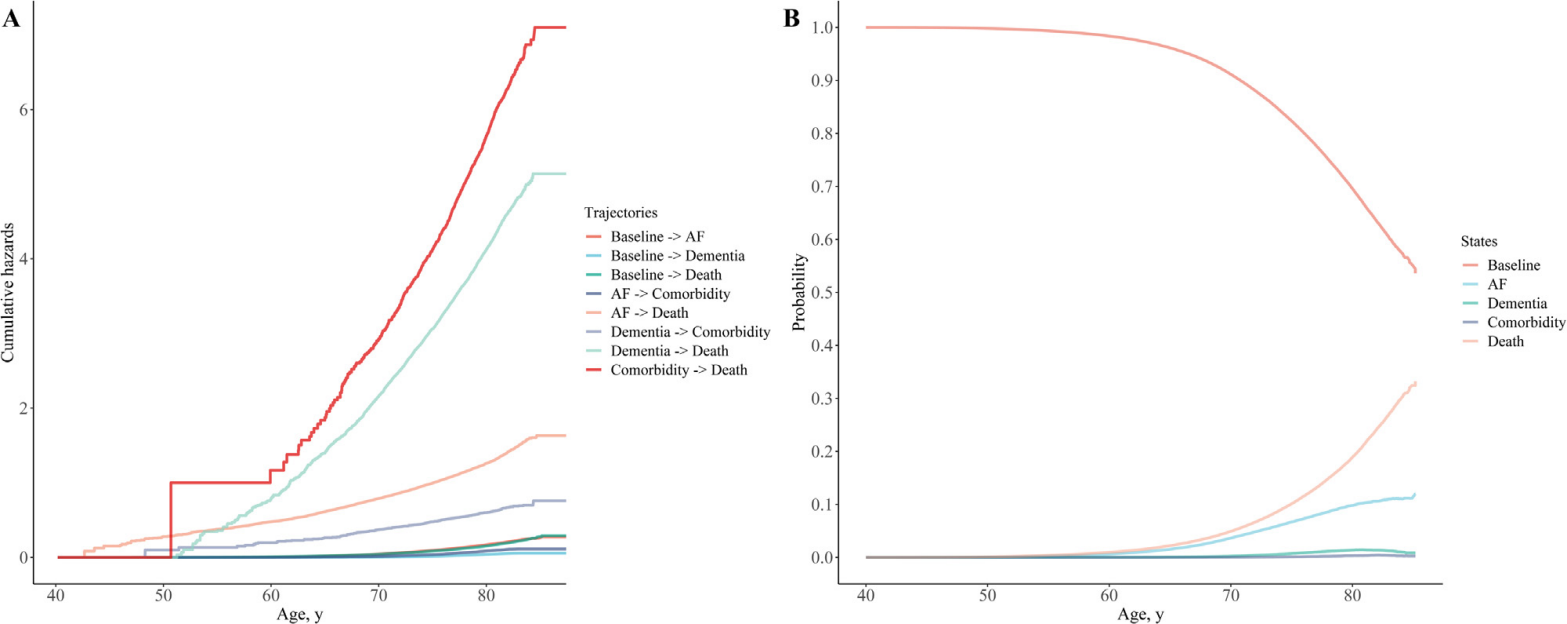
Cumulative hazards of disease trajectories and estimated state probabilities. AF, atrial fibrillation. (A) Cumulative hazards of observed trajectories of atrial fibrillation and dementia. (B) Estimated probabilities of predefined states.

As shown in Fig. 1, during a median follow-up of 13.82 (IQR, 13.09–14.54) years, a total of 28,612 (6.2 %) participants deceased without developing AF or dementia. A total of 30,807 (6.7 %) and 5719 (1.2 %) participants developed incident AF and dementia, respectively. Among the 30 807 participants with incident AF, 985 (3.2 %) participants further developed dementia and 6521 (21.2 %) participants died (Fig. 1). Among the 5719 participants with incident dementia, 370 (6.5 %) participants were further diagnosed with AF and 3029 (53.0 %) participants died (Fig. 1). A total of 1355 participants developed the combined comorbidity of AF and dementia during follow-up, among whom 837 (61.8 %) participants died (Fig. 1). Estimated cumulative hazards and state probabilities are presented in Fig. 2, showing the participants developing comorbidity of AF and dementia had the highest mortality hazard, followed by participants with incident dementia, and participants with incident AF (Fig. 2**A**). As shown in Fig. 2**B**, the probability of baseline (free of any pre-defined events) decreased with aging, while an inversed development pattern was observed in probabilities of death, developing AF, dementia, or comorbidity.

### 3.2 Associations between glycemic status with disease trajectories of af and dementia

**Fig. 3:**
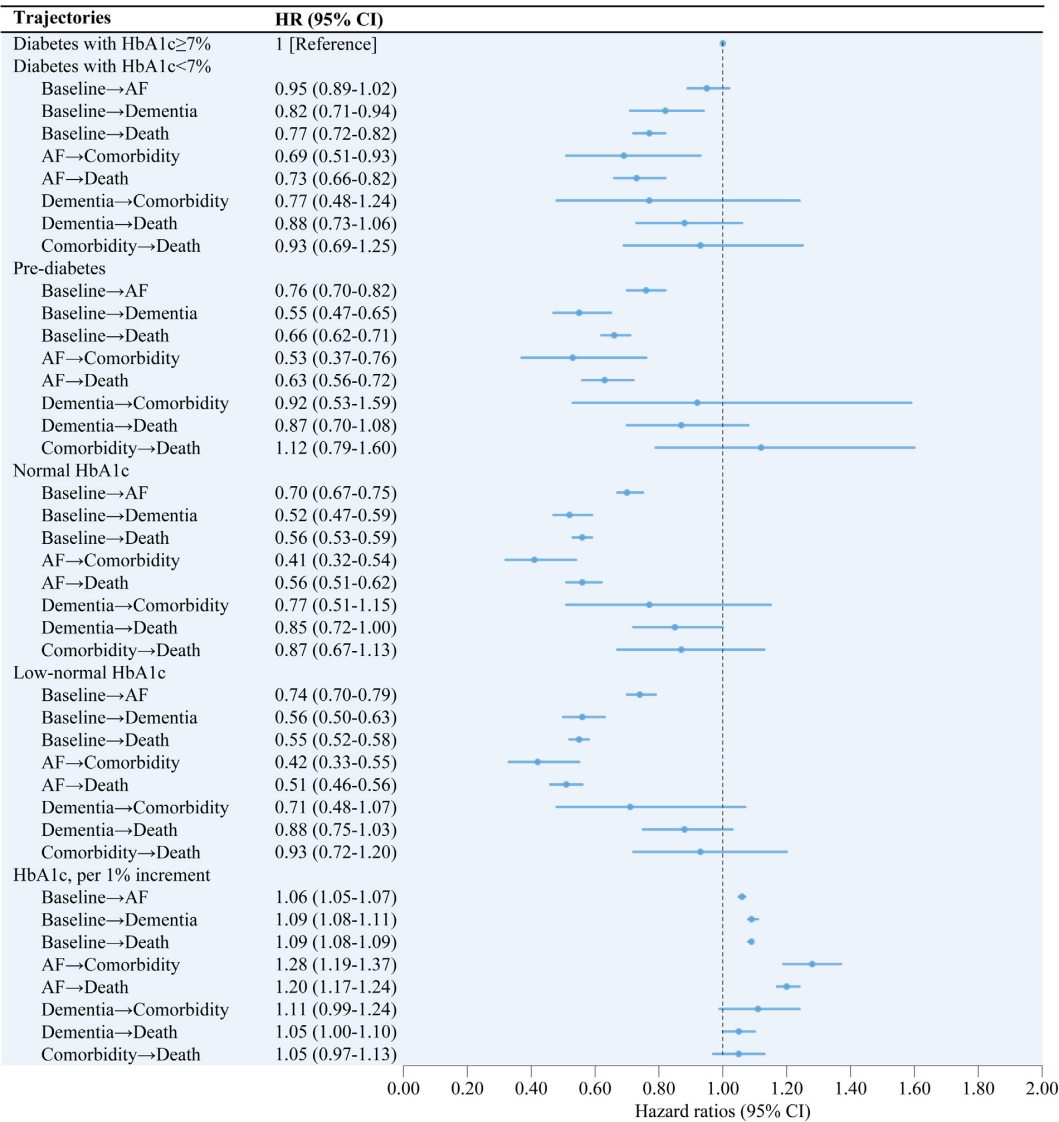
Associations between glycemic status and disease trajectories of atrial fibrillation and dementia. HbA1c, glycated hemoglobin; AF, atrial fibrillation; HR, hazard ratio; CI, confidence interval. Hazard ratios of associations between glycemic status with different trajectories of atrial fibrillation and dementia, controlling for sex, ethnicity, education, income, employment, alcohol consumption, physical activity, current smoking, chronic kidney disease, hypertension, and cardiovascular diseases.

As shown in Fig. 3, the better glycemic status was consistently associated with lower hazards of trajectories of AF and dementia, including trajectory from incident AF to comorbidity of AF and dementia. Compared with participants with diabetes with HbA1c≥7 %, those with diabetes with HbA1c <7 % had a 31 % lower hazard (HR, 0.69; 95 % CI, 0.51–0.93) of progression from incident AF to comorbidity and a 27 % lower hazard (HR, 0.73; 95 % CI, 0.66–0.82) of progression from incident AF to death (Fig. 3). Reduced hazards were also observed in trajectories from baseline to incident dementia and death when comparing diabetes with HbA1c<7 % and diabetes with HbA1c≥7 % (Fig. 3). Similar risk reductions were found in participants with pre-diabetes, normal HbA1c, and low-normal HbA1c, compared to diabetes with HbA1c≥7 %, except for the consistently observed lower hazard of development from baseline to incident AF (Fig. 3). Moreover, when analyzed as continuous variable, each 1 % increment in HbA1c was associated with increased hazards of most trajectories, particularly for trajectories from AF to comorbidity and from AF to death, with 28 % higher hazard (HR, 1.28; 95 % CI, 1.19–1.37) of development from AF to comorbidity and a 20 % higher hazard (HR, 1.20; 95 % CI, 1.17–1.24) of development from AF to death, respectively (Fig. 3).

**Fig. 4:**
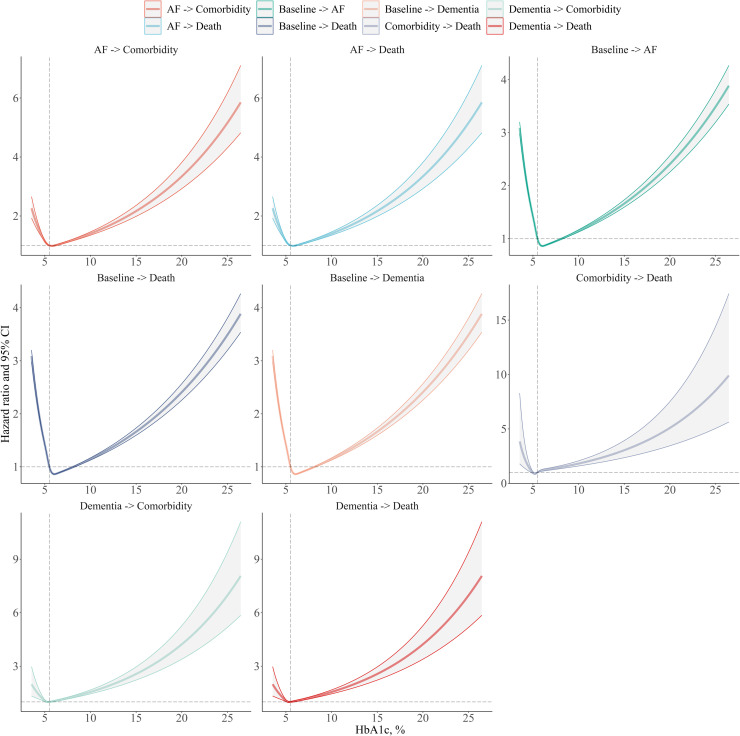
Dose-response curves of associations between HbA1c level and hazards of disease trajectories of atrial fibrillation and dementia. HbA1c, glycated hemoglobin; AF, atrial fibrillation; HR, hazard ratio; CI, confidence interval. Restricted cubic spline models were applied for depicting dose-response relationships, with four knots fixed at the 5th, 35th, 65th, and 95th percentiles. Solid lines represent point estimates and shadows represent 95 % confidence limits. Adjusted covariates included sex, ethnicity, education, income, employment, alcohol consumption, physical activity, current smoking, chronic kidney disease, hypertension, and cardiovascular diseases.

Dose-response relationships between HbA1c and trajectories hazards are presented in Fig. 4. A J-shaped association curve was broadly observed between the increased HbA1c level and elevated hazards of trajectories of AF and dementia.

### 3.3 Sensitivity analyses

Disease trajectories of AF with Alzheimer's disease and vascular dementia are presented in **Supplementary Figures 2** and **3**, respectively, with similar development patterns observed. As shown in **Supplementary Figure 4**, the better glycemic status was also associated with lower trajectories hazards of AF and Alzheimer's disease, with decreased risk of development from AF to comorbidity observed in participants of pre-diabetes, normal HbA1c, and low-normal HbA1c, compared to diabetes with HbA1c≥7 %. Each 1 % increased HbA1c was associated a 25 % higher hazard (HR, 1.25; 95 % CI, 1.11–1.42) of development from AF to comorbidity of Alzheimer's disease (**Supplementary Figure 4**). As shown in **Supplementary Figure 5**, similar association pattern was observed between better glycemic status and lower hazards of trajectories of AF and vascular dementia, except for a 49 % lower hazard (HR, 0.51; 95 % CI, 0.33–0.80) of development from incident AF to comorbidity observed in diabetes with HbA1c<7 %. Each 1 % increased HbA1c was associated a 42 % higher hazard (HR, 1.42; 95 % CI, 1.28–1.57) of development from AF to comorbidity of vascular dementia (**Supplementary Figure 5**). A consistent J-shaped curve was observed between HbA1c and hazards of trajectories of AF and Alzheimer's disease (**Supplementary Figure 6**), as well as trajectories of AF and vascular dementia (**Supplementary Figure 7**). Excluding prevalent cardiovascular disease cases did not bring substantial changes, except for null associations between diabetes with HbA1c<7 % and hazards of trajectories from AF to comorbidity (**Supplementary Figure 8**). Changing cut-off points of pre-diabetes did not bring substantial changes (**Supplementary Figure 9**), while simultaneously controlling for blood glucose and blood pressure moderately attenuated observed associations (**Supplementary Figure 10**). After further accounting for the condition where participants had the same AF and dementia diagnosis dates, similar disease trajectories and analysis results were observed, shown in **Supplementary Figures 11** and **12**. Further mediation analysis did not observe statistically significant mediation effect by incident AF in associations between glycemic status and dementia risk (**Supplementary Table 3**). Non-response analysis (**Supplementary Table 4**) showed that excluded participants, compared to included participants, were older, more likely to be men, non-white ethnicity, had lower socioeconomic positions, more prevalent chronic diseases, and higher HbA1c level.

## 4 Discussion

This large population-based prospective study found that the better glycemic status was consistently associated with decreased hazards of disease trajectories of AF and dementia, independently from other traditional risk factors. Additionally, we found even among individuals with diabetes, those with HbA1c<7 % had significantly lower hazard of progression from incident AF to comorbidity of dementia, compared to their counterparts with HbA1c≥7 %. A higher HbA1c was also associated with increased hazards of progression from incident AF to comorbidity of dementia or death. In addition, the glycemic status was consistently associated with disease trajectories of AF and sub-type dementia, including Alzheimer's disease and vascular dementia. The better glycemic status was associated with decreased hazards of trajectories from AF to comorbidity of AF and Alzheimer's disease/vascular dementia, with more pronounced protective associations observed in trajectories of AF and vascular dementia. These novel findings highlight the significance of reducing glycemic burden and reaching optimal glycemic status to prevent both AF onset and further development to comorbidity of dementia or death. To the best of our knowledge, the current study is the first one simultaneously investigating the associations between glycemic status with disease trajectories of AF and dementia, as well as its major subtypes.

Previous studies have reported a protective effect of the optimal glycemic status against onset of AF or dementia alone, without accounting for trajectories of the two diseases [10,12,13,[24], [25], [26], [27]]. A population-based case-control study found that prevalent diabetes and worse glycemic status were consistently associated with elevated AF risk [24]. Compared to non-diabetes participants, diabetes patients with HbA1c 7 %−8 % and HbA1c 8 %−9 % respectively had 48 % and 46 % higher odds of AF [24]. Another prospective study examined associations between type 2 diabetes and incident AF, showing that women with type 2 diabetes had 95 % (HR, 1.95; 95 % CI, 1.49–2.56) elevated risk of new onset of AF, compared to non-diabetes counterparts [25]. A study based on Swedish National Diabetes Registry observed significant associations between time-weighted mean HbA1c and elevated AF risk [26]. Compared to non-diabetes controls, individuals with diabetes and HbA1c≤6.9 % and HbA1c 7.0 %−7.8 % respectively had 24 % and 28 % higher risks of developing AF, respectively [26]. Previous studies also observed associations between better glycemic status with reduced dementia risk [12,13]. Another recent study evaluated prospective associations between longitudinal glycemic control pattern and dementia risk in type 2 diabetes patients [27], observing patients with more than 50 % of HbA1c measurements of less than 6 %, 6 % to less than 7 %, or 7 % to less than 8 % had significantly lower risks of dementia onset.

Aligning with these previous reports using the traditional analytical approach, our disease trajectory analysis showed that better glycemic status was consistently associated with decreased hazards of trajectories from baseline to incident AF or incident dementia. Moreover, we found that better glycemic status was persistently associated with a lower hazard of development from incident AF to comorbidity of AF and dementia, which has not been reported previously. We also observed strong dose-response associations between increased HbA1c level and elevated hazards of trajectories of AF and dementia, with each 1 % increment in HbA1c related to a 28 % higher hazard of development from incident AF to comorbidity of AF and dementia. In addition, the multi-state modeling approach enabled us to capture prospective associations between glycemic status and mortality hazard after developing AF, presenting more enriched insights into the importance of reaching optimal glycemic status to comprehensively improve the AF prognosis.

Accumulating evidence has indicated that individuals living with AF tend to have elevated risks of cognitive impairment and dementia [[28], [29], [30]]. On the other hand, people living with the comorbidity of AF and dementia constitute a special population, with more prevalent under-prescription of oral anticoagulants and higher long-term mortality risk [31,32]. Our findings also showed individuals developing comorbidity of AF and dementia had the highest mortality hazard, followed by those with incident dementia or AF alone. Hence, preventing development from AF to the comorbidity of AF and dementia is of huge significance, highlighting necessity of exploring applicable risk reduction strategies. And our study confirms the hypothesis regarding the role of optimal glycemic status in preventing disease trajectories of AF and dementia, which should be examined by further investigations.

In addition to the development from AF to comorbidity, we observed better glycemic status was consistently associated with a lower hazard of development from incident AF to mortality, which is interesting and relevant for current practice. It has been reported individuals with new-onset AF had a two-fold increased all-cause mortality risk compared to non-AF individuals [2]. Our analysis also showed that 21.2 % participants with incident AF deceased during follow-up, indicating the importance of alleviating excess mortality risk to further improve the prognosis of AF patients. Moreover, according to a previous study in type 2 diabetes individuals, the incident AF was associated with a 2.91-fold all-cause mortality and a 3.75-fold cardiovascular mortality, which further highlights the crucial importance of preventing post-AF mortality in diabetes population [33]. Consequently, our findings that diabetes individuals with HbA1c <7 % had a 27 % lower hazard of mortality post incident AF were clinically relevant. Such findings also add to the current literature by showing even in newly diagnosed AF patients with prevalent diabetes, strict glycemic control could be related with the improved long-term prognosis. Notably, the associations between better glycemic status and lower post-dementia mortality were largely insignificant, compared to the post-AF mortality. Although our analysis showed 53.0 % of participants with incident dementia deceased during follow-up, the null associations we observed did not support the benefits of strict glycemic control in alleviating dementia-related excess mortality risk. Further investigations are warranted to validate such findings.

We also evaluated disease trajectories of AF and major dementia subtypes, including Alzheimer's disease and vascular dementia. Intriguingly, associations between glycemic status with trajectories of AF and vascular dementia were more evident, compared to associations between glycemic status with trajectories of AF and Alzheimer's disease. Even among individuals with diagnosed diabetes, those with HbA1c<7 %, compared to their counterparts with HbA1c≥7 %, not only had a 37 % lower hazard of developing vascular dementia, but a 49 % reduction in risk of development from incident AF to comorbidity of vascular dementia. By contrast, better glycemic status in individuals with diabetes was not associated with reduced risk of development from incident AF to comorbidity of Alzheimer's disease. A strong dose-response association was also observed between HbA1c and trajectories of AF and vascular dementia, with each 1 % increment in HbA1c related to a 42 % higher hazard of development from incident AF to comorbidity of vascular dementia. Nevertheless, as the dementia ascertainment algorithm has a relatively low positive predictive value for vascular dementia (only 33.3 %), cautions should be taken when interpreting results regarding different etiologies of dementia. Further investigations are therefore warranted to confirm our findings.

Compared to individuals with diabetes and HbA1c≥7 %, we found pre-diabetes participants respectively had a 47 % lower hazard of development from AF to comorbidity of dementia, approximating risk reductions associated with normal and low-normal HbA1c. Considering the well-established evidence of cardiovascular benefits of preventing or delaying new-onset diabetes among pre-diabetes individuals [34], our findings provide novel clues indicating the significance in achieving substantial risk reductions in disease trajectories of AF and dementia.

Compared with previous studies, our study adds important new evidence for benefits of reaching optimal glycemic status in preventing the onset and improving prognosis of AF and dementia, by accounting for trajectories of the two diseases. Notably, the associations between glycemic status and development from AF to comorbidity of dementia persisted after excluding individuals with prevalent cardiovascular diseases. Collectively, these findings highlight the importance of maintaining optimal glycemic status, for people managed in the current practice to prevent serious comorbidities.

Our study possesses several strengths. First, due to the large sample-size and long-term follow-up, we were able to comprehensively evaluate the dynamic disease trajectories of AF and dementia, as well as assess the role of glycemic status in observed trajectories. Second, we additionally accounted for disease trajectories of AF and major dementia subtypes, extending the significance of our findings. Finally, several sensitivity analyses were conducted, supporting the robustness of major findings.

## 5 Limitations

This study also has limitations. First, the dementia ascertainment procedure might underestimate the true number of dementia cases. Despite that the outcome algorithm has been previously validated, the lack of primary records poses challenges for timely and accurate ascertainment of dementia cases. Moreover, although we have incorporated death certificates and surgical operation records for identifying incident AF cases, it was possible that some AF patients were not diagnosed until death, indicating the challenge of underdiagnosed AF. Second, lack of repeated HbA1c measurements prevented us from capturing the longitudinal glycemic status pattern and evaluating the value in disease trajectories prevention. Third, most of the UK Biobank participants were of white ethnicity, hence restricting the generalization of our findings to other ethnicities. Finally, the failure of precluding residual confounding prevents us from making formal conclusions regarding the causality of observed associations.

## 6 Conclusions

In conclusion, we found that better glycemic status was consistently associated with decreased hazards of disease trajectories of AF and dementia, including the lower hazard of progression from incident AF to comorbidity of AF and dementia. These findings underscore the significance of reaching optimal glycemic status to alleviate the huge disease burden of both AF and dementia simultaneously. Further investigations capable of making causal inference are warranted to confirm our findings.

## CRediT authorship contribution statement

**Chenglong Li:** Writing – original draft, Conceptualization. **Daijun He:** Writing – review & editing. **Yufan Liu:** Writing – review & editing. **Chao Yang:** Writing – review & editing. **Luxia Zhang:** Writing – review & editing, Supervision, Funding acquisition, Formal analysis, Data curation. **Rodica Pop-Busui:** Writing – review & editing.

## Declaration of competing interest

The authors declare the following financial interests/personal relationships which may be considered as potential competing interests:

Luxia Zhang reports financial support was provided by National Key Research and Development Program of China. Luxia Zhang reports financial support was provided by National Natural Science Foundation of China. Luxia Zhang reports financial support was provided by Chinese Scientific and Technical Innovation Project 2030. Luxia Zhang reports financial support was provided by CAMS Innovation Fund for Medical Sciences. Luxia Zhang reports financial support was provided by PKU-Baidu Fund. Chenglong Li reports financial support was provided by Postdoctoral Fellowship Program of CPSF. If there are other authors, they declare that they have no known competing financial interests or personal relationships that could have appeared to influence the work reported in this paper.

## Data Availability

The data that support the findings of this study are available from the UK Biobank project site, subject to registration and application process. Further details can be found at ukbiobank.ac.uk

## Appendix Supplementary materials

Image, application 1
