# Supplementary material for: Associations of glycemic status with dynamic disease trajectories of atrial fibrillation and dementia
Source: J Prev Alzheimers Dis. 2025 Jan 13;12(3):100047. doi: 10.1016/j.tjpad.2024.100047 (PMC12183936; doi:10.1016/j.tjpad.2024.100047)
Supplement: Supplementary file 1 [file mmc1.docx]

**SUPPLEMENTAL MATERIAL**

**Supplementary tables and figures**

[**Supplementary Table 1.** Definition and assessment of atrial fibrillation in UK Biobank.](#_Toc183199422)

[**Supplementary Table 2.** ICD-9 and ICD-10 codes used to ascertain all-cause and sub-type dementia cases.](#_Toc183199423)

[**Supplementary Table 3.** Mediation analysis assessing the mediation effect of incident atrial fibrillation in associations between glycemic status and dementia risk.](#_Toc183199424)

[**Supplementary Table 4.** Non-response analysis comparing baseline characteristics of included and excluded participants in UK Biobank.](#_Toc183199425)

**Supplementary Figure 1.** Participants selection diagram.

**Supplementary Figure 2.** Disease trajectories of atrial fibrillation and Alzheimer’s disease.

**Supplementary Figure 3.** Disease trajectories of atrial fibrillation and vascular dementia.

**Supplementary Figure 4.** Associations between glycemic status and disease trajectories of atrial fibrillation and Alzheimer’s disease.

**Supplementary Figure 5.** Associations between glycemic status and disease trajectories of atrial fibrillation and vascular dementia.

**Supplementary Figure 6.** Dose-response curves of associations between HbA1c level and hazards of disease trajectories of atrial fibrillation and Alzheimer’s disease.

**Supplementary Figure 7.** Dose-response curves of associations between HbA1c level and hazards of disease trajectories of atrial fibrillation and vascular dementia.

**Supplementary Figure 8.** Associations between glycemic status and disease trajectories of atrial fibrillation and dementia, further excluding individuals with prevalent cardiovascular disease.

**Supplementary Figure 9.** Associations between glycemic status and disease trajectories of atrial fibrillation and dementia, changing the criteria of pre-diabetes per ADA guidelines.

**Supplementary Figure 10.** Associations between glycemic status and disease trajectories of atrial fibrillation and dementia, further controlling for values of blood glucose and blood pressure.

**Supplementary Figure 11.** Disease trajectories of atrial fibrillation and dementia, further including participants with same diagnosed dates of atrial fibrillation and dementia and therefore accounting for the transition from baseline to comorbidity.

**Supplementary Figure 12.** Associations between glycemic status and disease trajectories of atrial fibrillation and dementia, further including participants with same diagnosed dates of atrial fibrillation and dementia and therefore accounting for the transition from baseline to comorbidity.

**Supplementary Table 1.** Definition and assessment of atrial fibrillation in UK Biobank.

| **Approach** | **Assessment** | **UK Biobank Data-Field ID** |
| --- | --- | --- |
| ICD-10 codes (incorporating data from inpatient records and death registry) | Codes I48, I48.1, I48.2, I48.3, I48.4, I48.9 | 131350, 131351 |
| OPCS4 codes | Codes K50.1, K62.2, K62.3, K62.4 | 41272, 41282 |
| Self-report of history of cardiovascular disease | Verbal interview for non-cancer illness | 20002 |

**Supplementary Table 2.** ICD-9 and ICD-10 codes used to ascertain all-cause and sub-type dementia cases.

| **ICD-9 code** | | | | |
| --- | --- | --- | --- | --- |
| ICD-9 code | ICD-9 text | Alzheimer’s disease | Vascular dementia | All-cause dementia |
| 290.2 | Senile dementia, depressed or paranoid type |  |  | √ |
| 290.3 | Senile dementia with acute confusional state |  |  | √ |
| 290.4 | Arteriosclerotic dementia |  | √ | √ |
| 291.2 | Other alcoholic dementia |  |  | √ |
| 294.1 | Dementia in other conditions classified elsewhere |  |  | √ |
| 331.0 | Alzheimer's disease | √ |  | √ |
| 331.1 | Pick's disease |  |  | √ |
| 331.2 | Senile degeneration of brain |  |  | √ |
| 331.5 | Creutzfeldt-Jakob disease |  |  | √ |
| **ICD-10 code** | | | | |
| ICD-10 code | ICD-10 text | Alzheimer’s disease | Vascular dementia | All-cause dementia |
| A81.0 | Sporadic Creutzfeldt-Jakob disease |  |  | √ |
| F00 | Dementia in Alzheimer's disease | √ |  | √ |
| F00.0 | Dementia in Alzheimer's disease with early onset | √ |  | √ |
| F00.1 | Dementia in Alzheimer's disease with late onset | √ |  | √ |
| F00.2 | Dementia in Alzheimer's disease, atypical or mixed type | √ |  | √ |
| F00.9 | Dementia in Alzheimer's disease, unspecified | √ |  | √ |
| F01 | Vascular dementia |  | √ | √ |
| F01.0 | Vascular dementia of acute onset |  | √ | √ |
| F01.1 | Multi-infarct dementia |  | √ | √ |
| F01.2 | Subcortical vascular dementia |  | √ | √ |
| F01.3 | Mixed cortical and sub-cortical vascular dementia |  | √ | √ |
| F01.8 | Other vascular dementia |  | √ | √ |
| F01.9 | Vascular dementia, unspecified |  | √ | √ |
| F02 | Dementia in other diseases classified elsewhere |  |  | √ |
| F02.0 | Dementia in Picks disease |  |  | √ |
| F02.1 | Dementia in Creutzfeldt-Jacob disease |  |  | √ |
| F02.2 | Dementia in Huntington’s disease |  |  | √ |
| F02.3 | Dementia in Parkinson’s disease |  |  | √ |
| F02.4 | Dementia in HIV disease |  |  | √ |
| F02.8 | Dementia in other specified diseases classified elsewhere |  |  | √ |
| F03 | Unspecified dementia |  |  | √ |
| F05.1 | Delirium superimposed on dementia |  |  | √ |
| F10.6 | Mental and behavioural disorders due to use of alcohol - amnesic syndrome |  |  | √ |
| G30 | Alzheimer’s disease | √ |  | √ |
| G30.0 | Alzheimer’s disease with early onset | √ |  | √ |
| G30.1 | Alzheimer’s disease with late onset | √ |  | √ |
| G30.8 | Other Alzheimer's disease | √ |  | √ |
| G30.9 | Alzheimer's disease unspecified | √ |  | √ |
| G31.0 | Circumscribed brain atrophy |  |  | √ |
| G31.1 | Senile degeneration of brain |  |  | √ |
| G31.8 | Other specified degenerative diseases of  nervous system |  |  | √ |
| I67.3 | Binswanger’s disease |  | √ |  |

**Supplementary Table 3.** Mediation analysis assessing the mediation effect of incident atrial fibrillation in associations between glycemic status and dementia risk.

| **Glycemic status** | **Hazard ratio (95% CI)** | | **Proportion mediated (%) ^a^**  **(95% CI)** |
| --- | --- | --- | --- |
|  | **Unadjusted for AF** | **Adjusted for AF** |  |
| **All-cause dementia** | | | |
| HbA1c, per 1% increment | 1.10 (1.06 to 1.14) | 1.10 (1.06 to 1.14) | NA ^b^ |
| **Alzheimer’s disease** | | | |
| HbA1c, per 1% increment | 1.10 (1.06 to 1.14) | 1.10 (1.06 to 1.14) | NA ^b^ |
| **Vascular dementia** | | | |
| HbA1c, per 1% increment | 1.13 (1.08 to 1.17) | 1.13 (1.08 to 1.17) | NA ^b^ |

HbA1c, glycated hemoglobin; AF, atrial fibrillation; CI, confidence interval.

^a^ Mediation proportion was calculated using the difference method, by comparing effect estimates before and after adjustment of hypothesized mediator (e.g. incident AF). Adjusted covariates included age, sex, ethnicity, education, income, employment, alcohol consumption, physical activity, current smoking, chronic kidney disease, hypertension, and cardiovascular diseases.

^b^ Mediation proportion was not calculated as no attenuated association was observed after controlling for the hypothesized mediator (e.g. the proportion being mediated was too small to be calculated reliably).

**Supplementary Table 4.** Non-response analysis comparing baseline characteristics of included and excluded participants in UK Biobank.

| **Characteristics ^a^** | **Excluded**  **N=44 001** | **Included**  **N=** **458 368** | ***P* ^b^** |
| --- | --- | --- | --- |
| Age, mean (SD), years | 57.4 (8.1) | 56.4 (8.1) | <0.001 |
| Men | 21 116 (48.0) | 207 952 (45.4) | <0.001 |
| White ethnicity | 39 385 (89.5) | 432 619 (94.4) | <0.001 |
| Higher education | 18 393 (41.8) | 215 121 (46.9) | <0.001 |
| Annual income ≥ £31 000 | 15 774 (35.8) | 204 138 (44.5) | <0.001 |
| Employed | 39 422 (89.6) | 420 758 (91.8) | <0.001 |
| Current smoking | 4495 (10.2) | 48 466 (10.6) | <0.001 |
| Alcohol intake once per week | 28 908 (65.7) | 317 510 (69.3) | <0.001 |
| Physical activity ≥ 150 min/week | 27 791 (63.2) | 328 497 (71.7) | <0.001 |
| Chronic kidney disease | 1849 (4.2) | 12 131 (2.6) | <0.001 |
| Hypertension | 25 980 (59.0) | 254 447 (55.5) | <0.001 |
| Diabetes | 3423 (7.8) | 28 219 (6.2) | <0.001 |
| Cardiovascular disease | 10 850 (24.7) | 29 502 (6.4) | <0.001 |
| HbA1c, median (IQR), % | 5.5 (5.3-5.8) | 5.4 (5.1-5.6) | <0.001 |

^a^ Data represented characteristics as mean (SD) or median (IQR) or n (%).

^b^ P value reported for differences between groups using t test or chi-square test or Wilcoxon rank sum test.

HbA1c, glycated hemoglobin


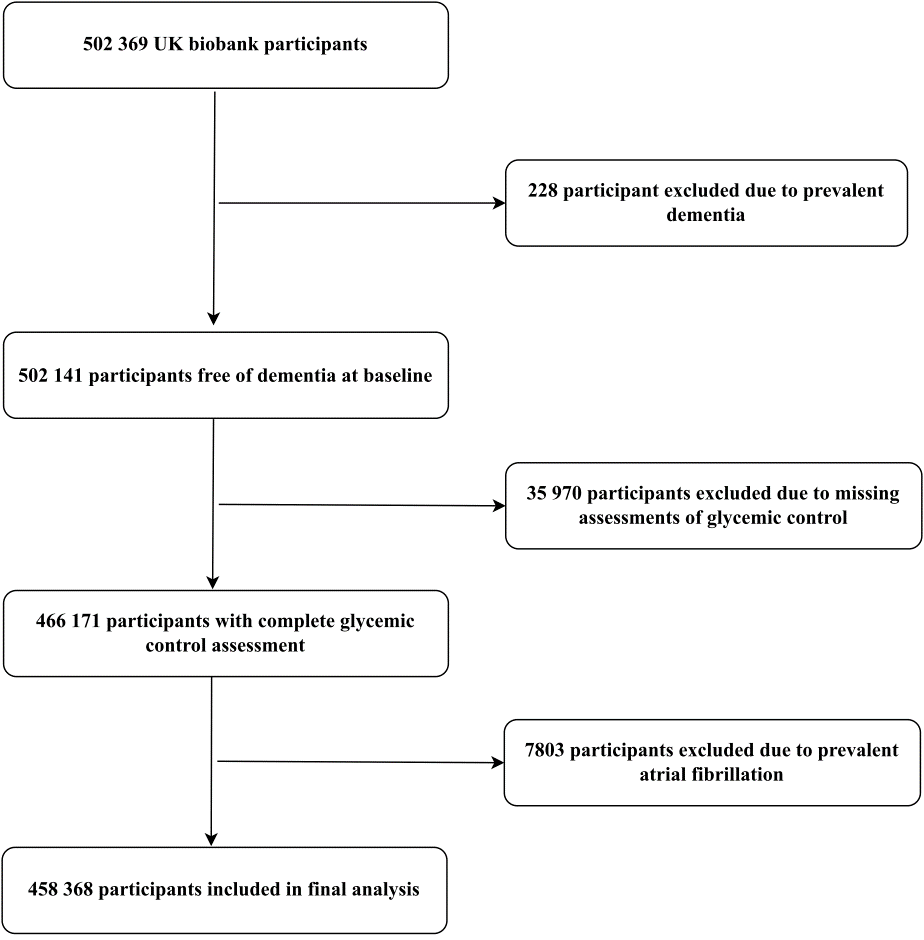


**Supplementary Figure 1.** Participants selection diagram.


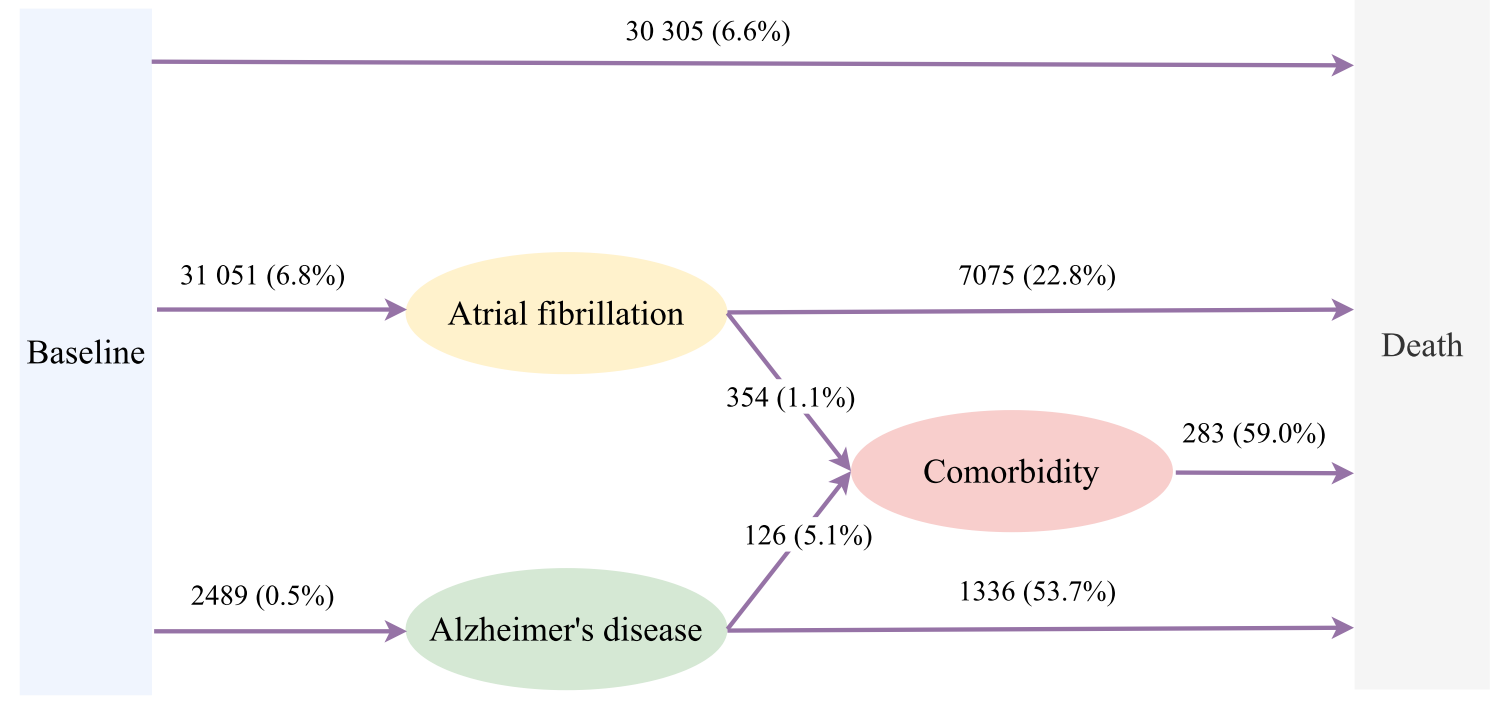


**Supplementary Figure 2.** Disease trajectories of atrial fibrillation and Alzheimer’s disease.

Observed trajectories (expressed as numbers and percentages of participants in the previous stage) of atrial fibrillation and Alzheimer’s disease.


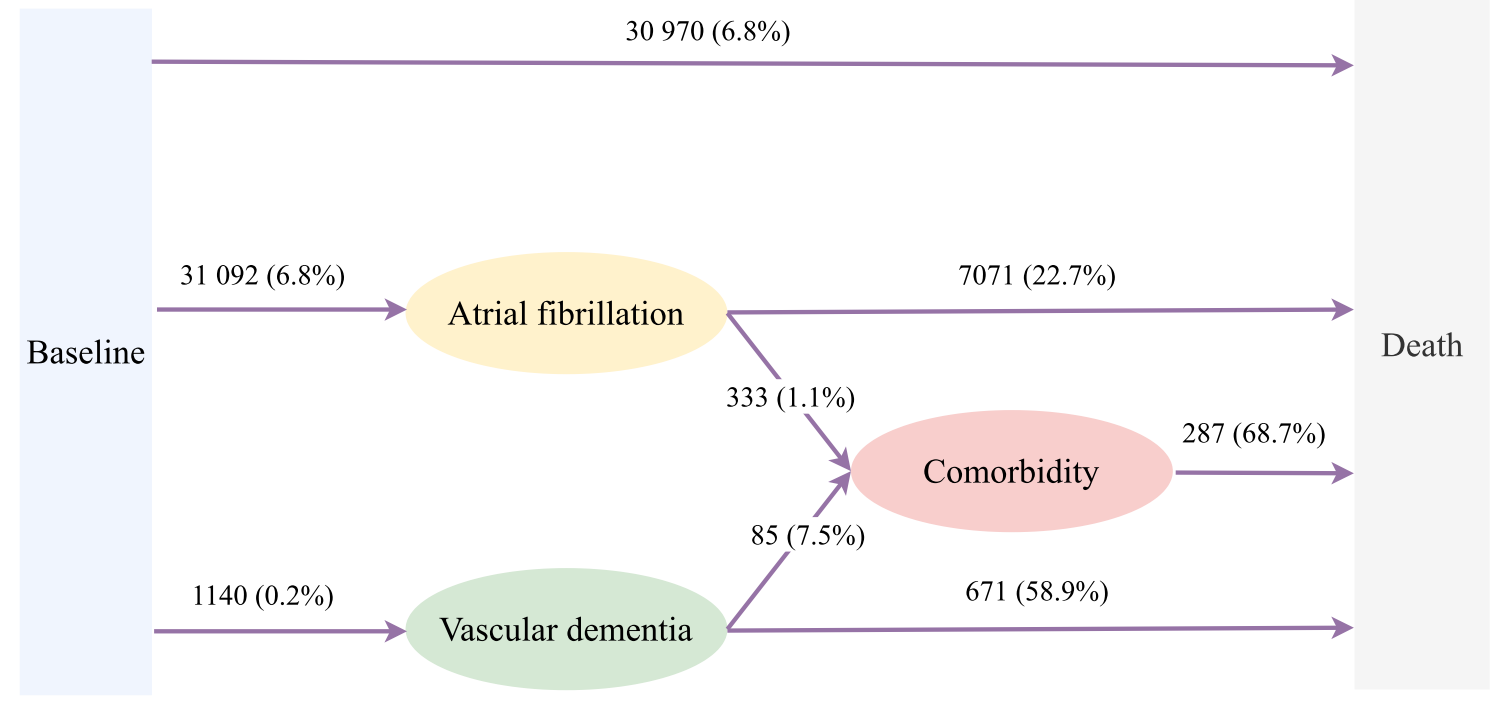


**Supplementary Figure 3.** Disease trajectories of atrial fibrillation and vascular dementia.

Observed trajectories (expressed as numbers and percentages of participants in the previous stage) of atrial fibrillation and vascular dementia.


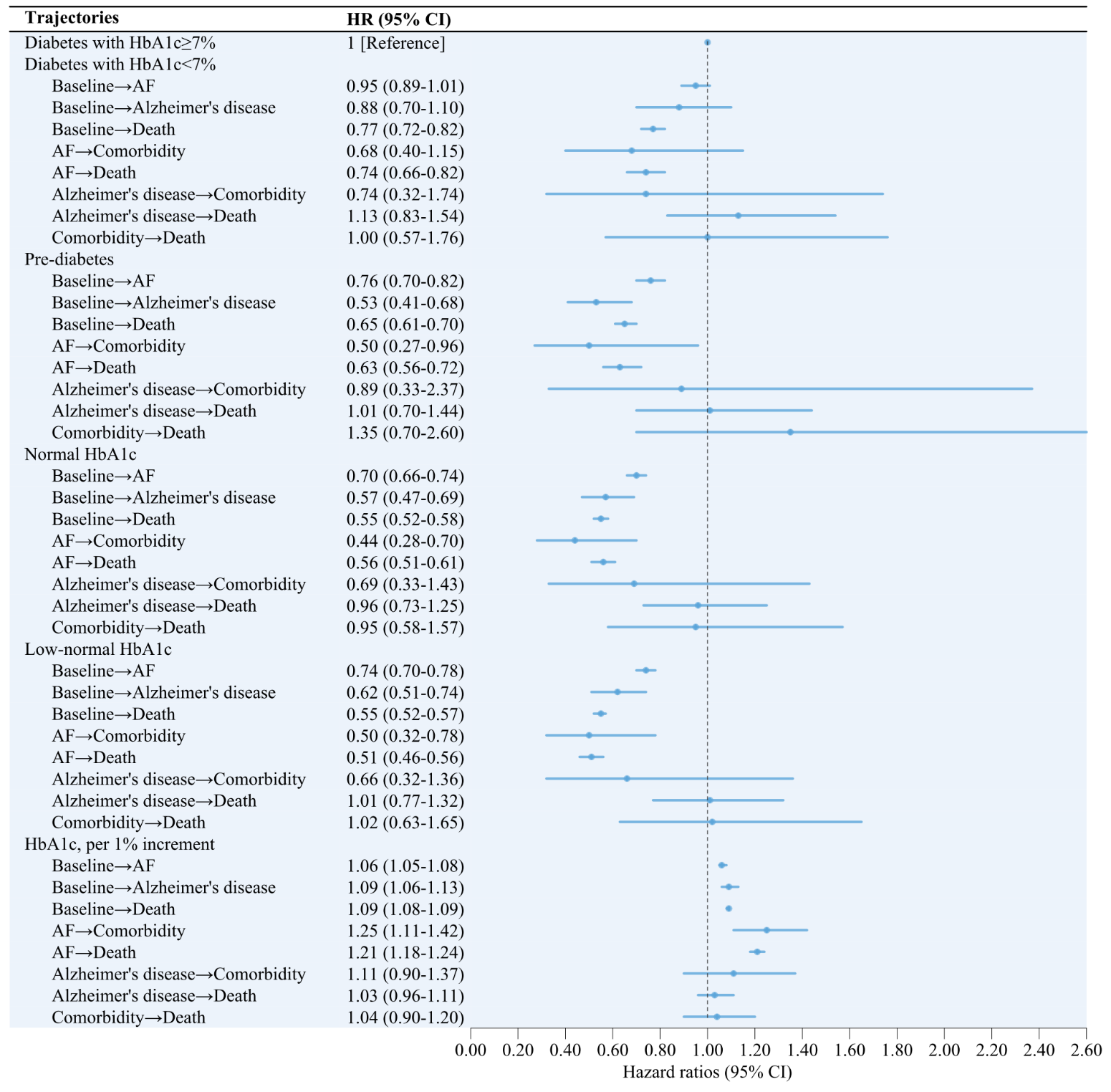
**Supplementary Figure 4.** Associations between glycemic status and disease trajectories of atrial fibrillation and Alzheimer’s disease.

HbA1c, glycated hemoglobin; AF, atrial fibrillation; HR, hazard ratio; CI, confidence interval.

Hazard ratios of associations between glycemic status with different trajectories of atrial fibrillation and Alzheimer’s disease, controlling for sex, ethnicity, education, income, employment, alcohol consumption, physical activity, current smoking, chronic kidney disease, hypertension, and cardiovascular diseases.


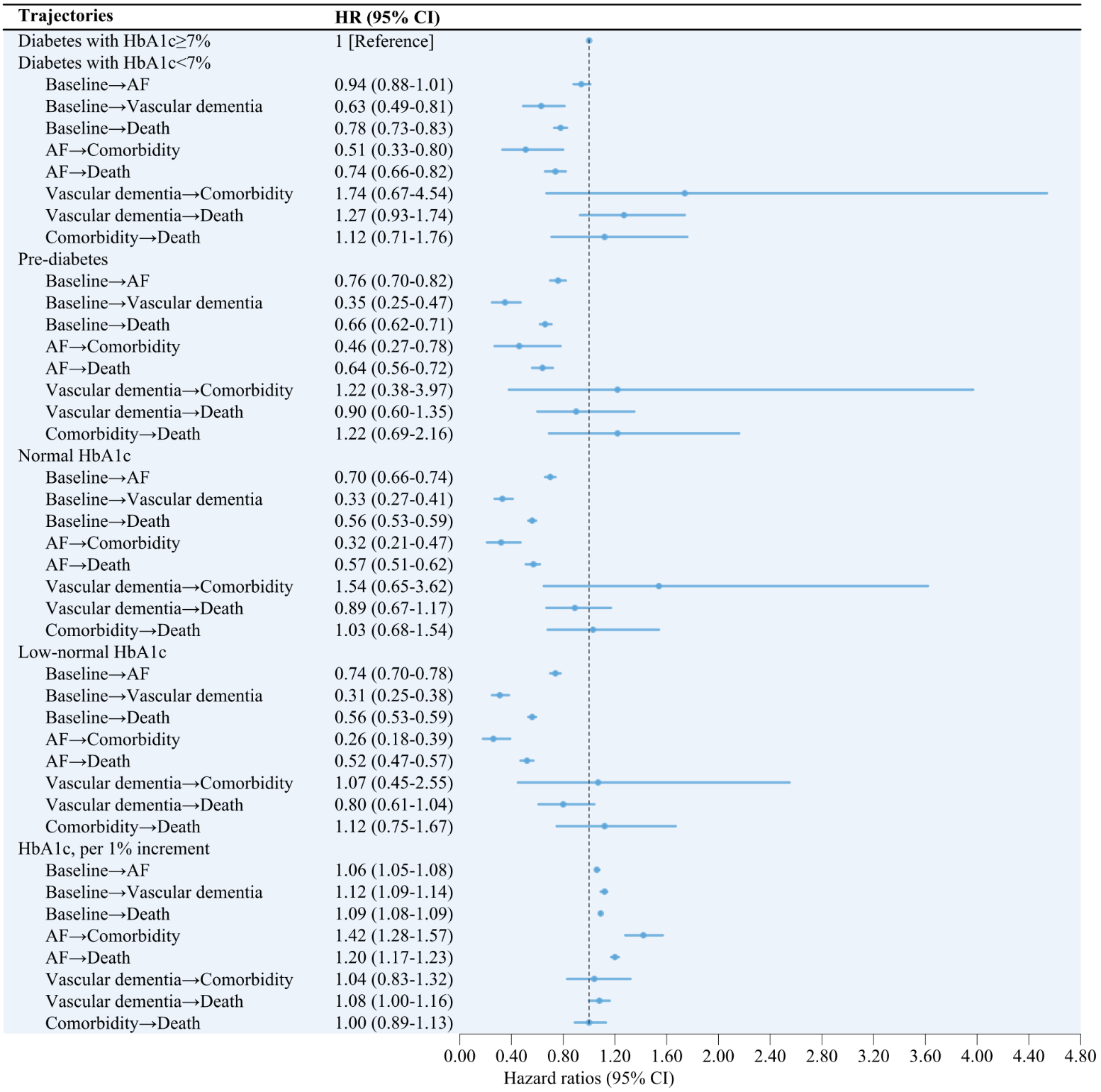


**Supplementary Figure 5.** Associations between glycemic status and disease trajectories of atrial fibrillation and vascular dementia.

HbA1c, glycated hemoglobin; AF, atrial fibrillation; HR, hazard ratio; CI, confidence interval.

Hazard ratios of associations between glycemic status with different trajectories of atrial fibrillation and vascular dementia, controlling for sex, ethnicity, education, income, employment, alcohol consumption, physical activity, current smoking, chronic kidney disease, hypertension, and cardiovascular diseases.


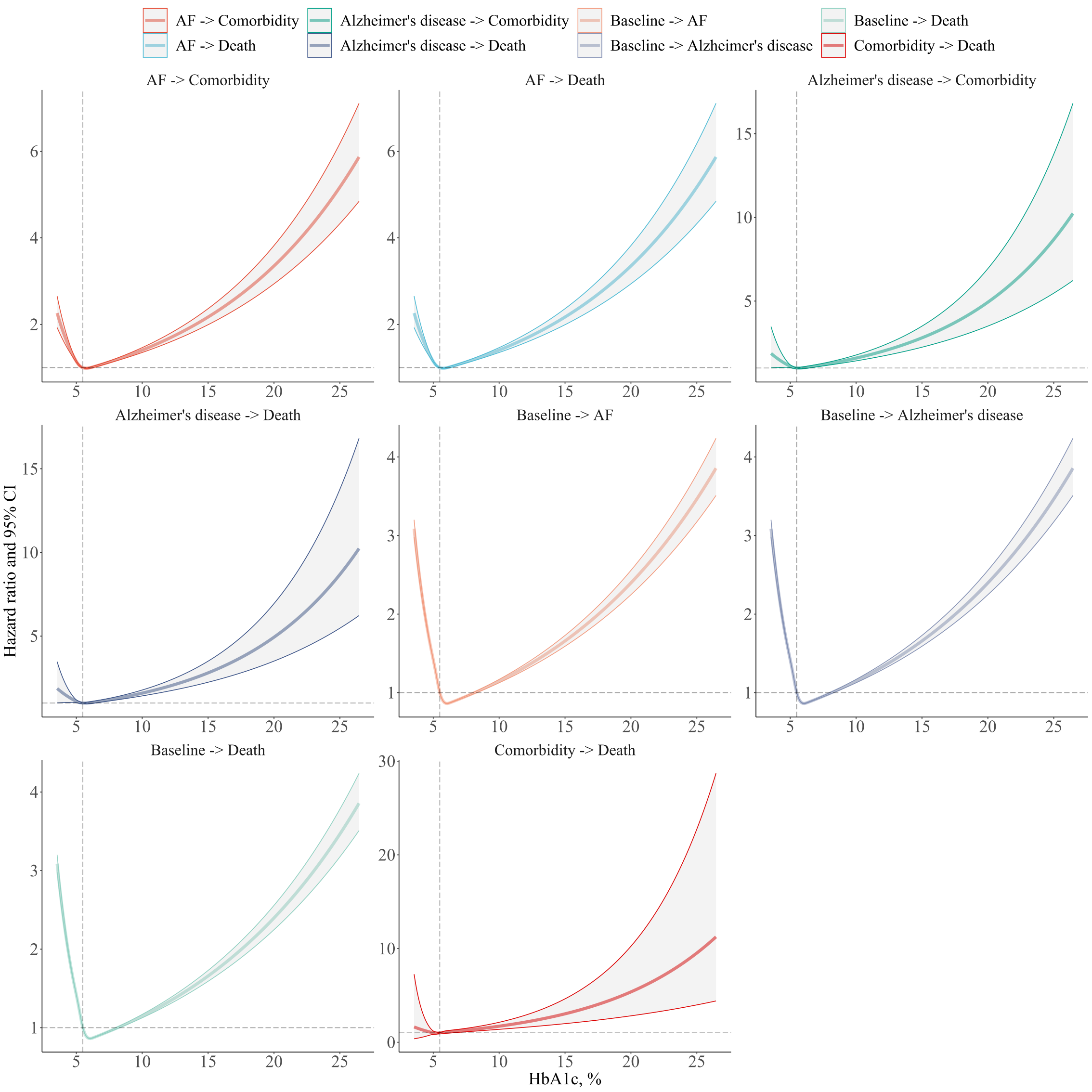


**Supplementary Figure 6.** Dose-response curves of associations between HbA1c level and hazards of disease trajectories of atrial fibrillation and Alzheimer’s disease.

HbA1c, glycated hemoglobin; AF, atrial fibrillation; HR, hazard ratio; CI, confidence interval.

Restricted cubic spline models were applied for depicting dose-response relationships, with four knots fixed at the 5th, 35th, 65th, and 95th percentiles. Solid lines represent point estimates and shadows represent 95% confidence limits. Adjusted covariates included sex, ethnicity, education, income, employment, alcohol consumption, physical activity, current smoking, chronic kidney disease, hypertension, and cardiovascular diseases.


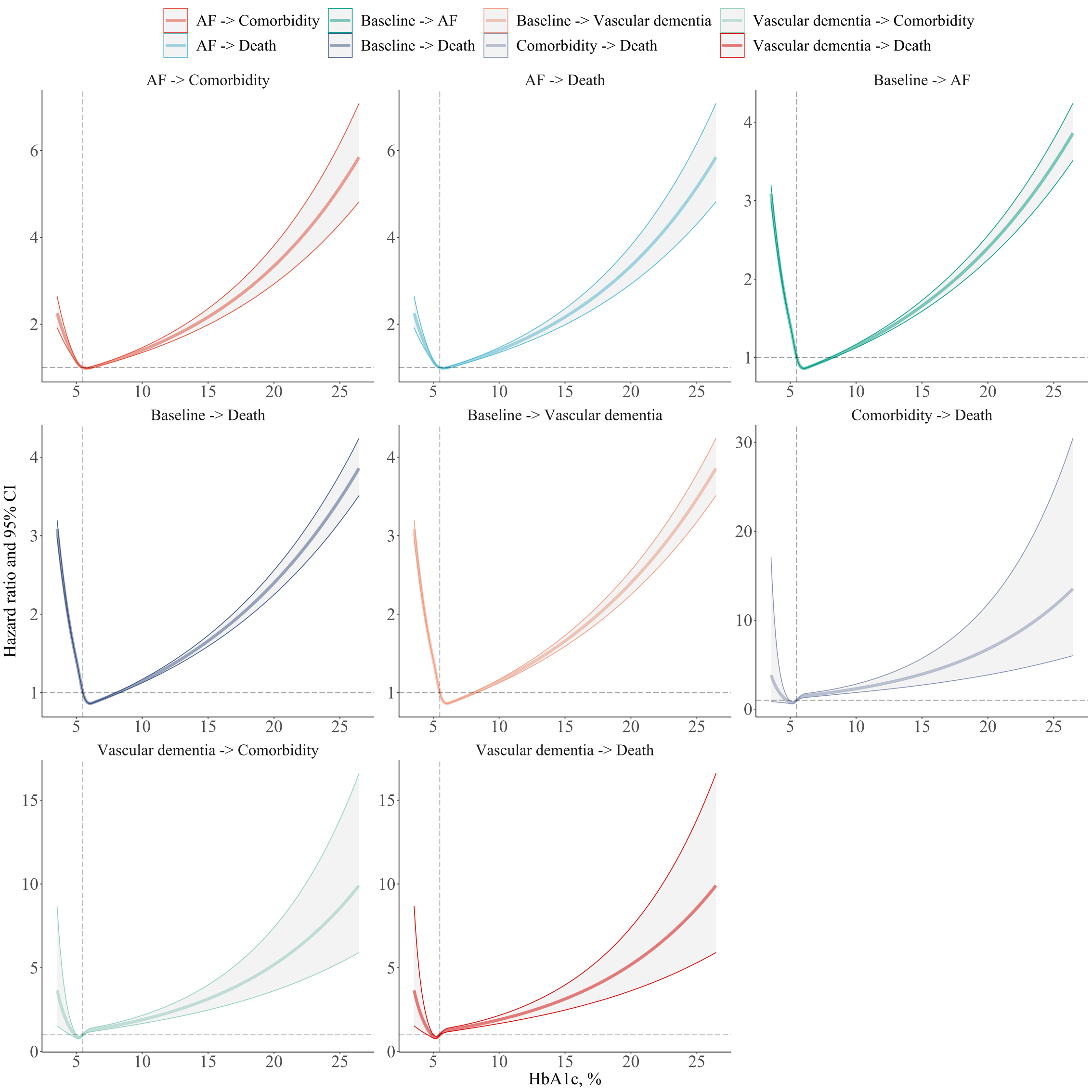


**Supplementary Figure 7.** Dose-response curves of associations between HbA1c level and hazards of disease trajectories of atrial fibrillation and vascular dementia.

HbA1c, glycated hemoglobin; AF, atrial fibrillation; HR, hazard ratio; CI, confidence interval.

Restricted cubic spline models were applied for depicting dose-response relationships, with four knots fixed at the 5th, 35th, 65th, and 95th percentiles. Solid lines represent point estimates and shadows represent 95% confidence limits. Adjusted covariates included sex, ethnicity, education, income, employment, alcohol consumption, physical activity, current smoking, chronic kidney disease, hypertension, and cardiovascular diseases.


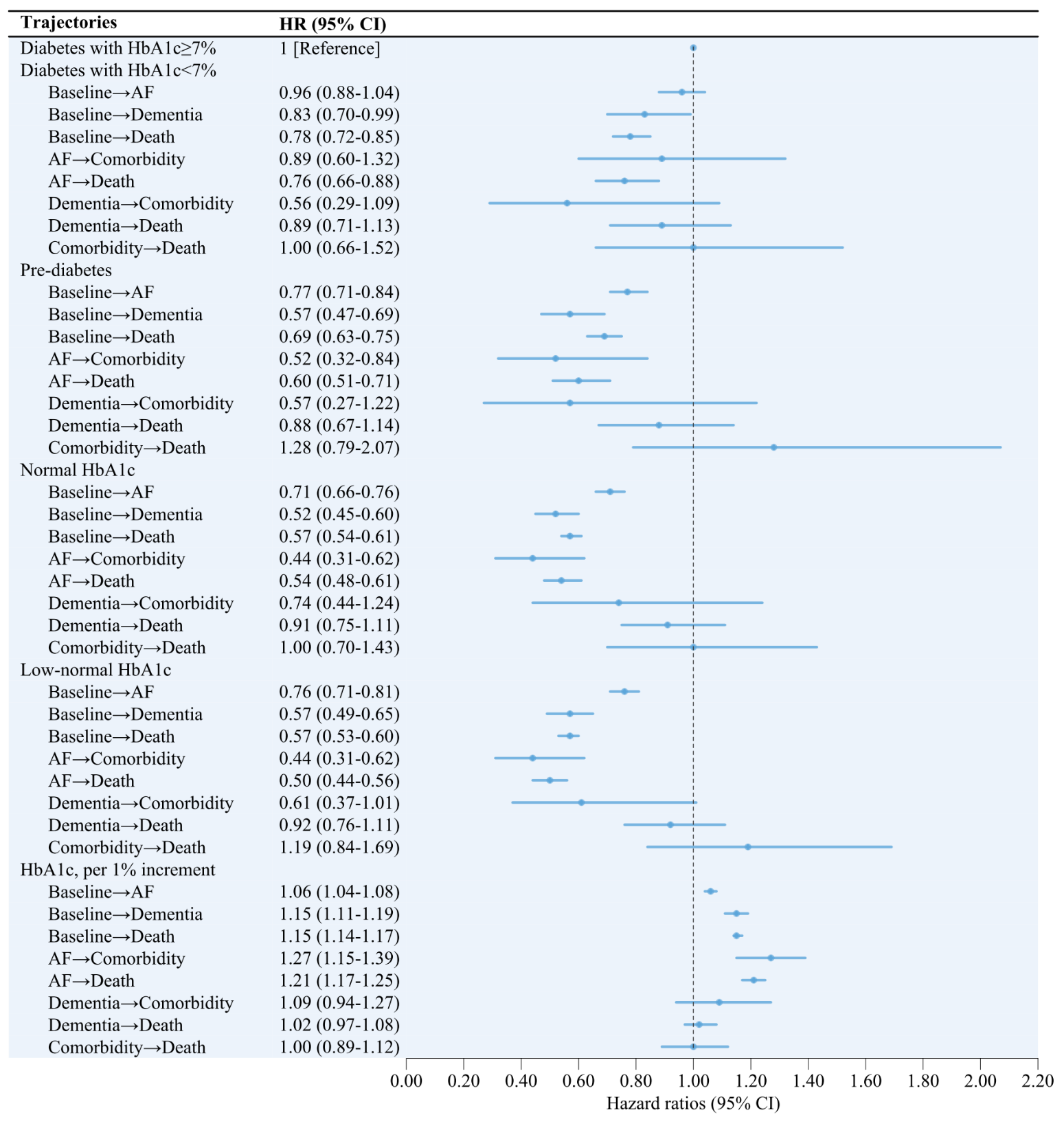
 **Supplementary Figure 8.** Associations between glycemic status and disease trajectories of atrial fibrillation and dementia, further excluding individuals with prevalent cardiovascular disease.

HbA1c, glycated hemoglobin; AF, atrial fibrillation; HR, hazard ratio; CI, confidence interval.

Hazard ratios of associations between glycemic status with different trajectories of atrial fibrillation and dementia, controlling for sex, ethnicity, education, income, employment, alcohol consumption, physical activity, current smoking, chronic kidney disease, and hypertension.


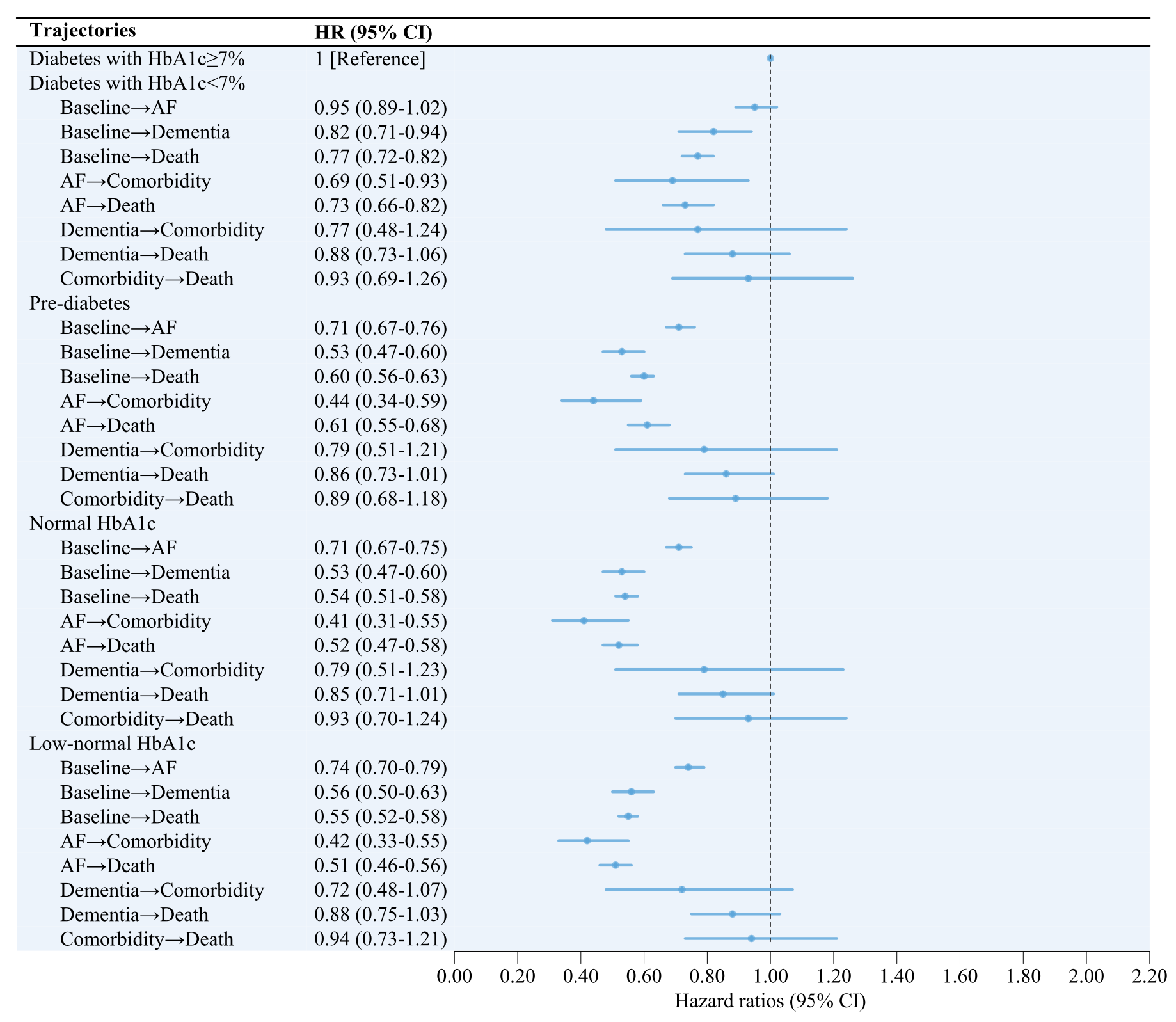


**Supplementary Figure 9.** Associations between glycemic status and disease trajectories of atrial fibrillation and dementia, changing the criteria of pre-diabetes per ADA guidelines.

HbA1c, glycated hemoglobin; AF, atrial fibrillation; HR, hazard ratio; CI, confidence interval.

Hazard ratios of associations between glycemic status with different trajectories of atrial fibrillation and dementia, controlling for sex, ethnicity, education, income, employment, alcohol consumption, physical activity, current smoking, chronic kidney disease, and hypertension. Pre-diabetes was defined as the HbA1c of 5.7-6.4%.


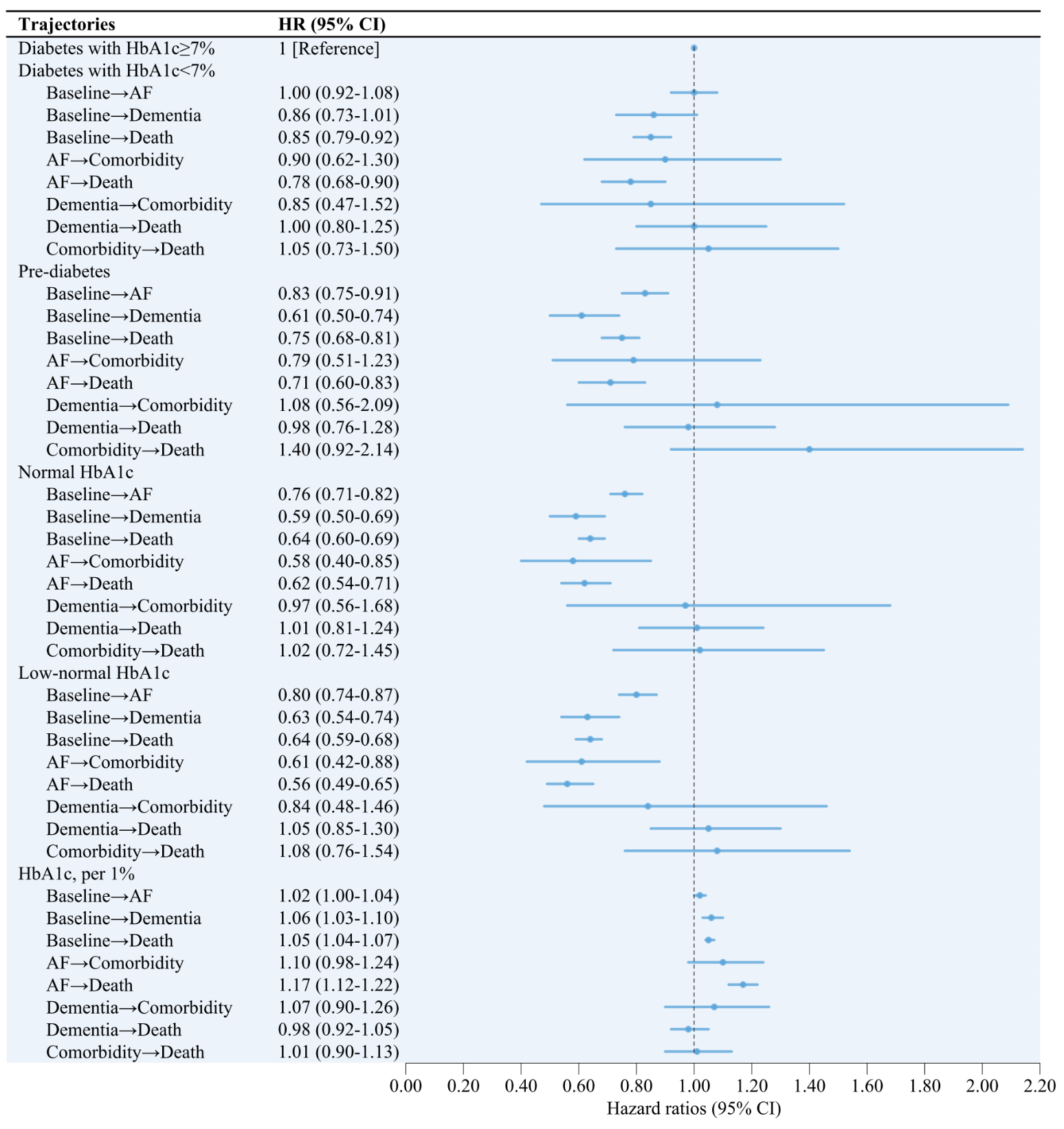
 **Supplementary Figure 10.** Associations between glycemic status and disease trajectories of atrial fibrillation and dementia, further controlling for values of blood glucose and blood pressure.

HbA1c, glycated hemoglobin; AF, atrial fibrillation; HR, hazard ratio; CI, confidence interval.

Hazard ratios of associations between glycemic status with different trajectories of atrial fibrillation and dementia, controlling for sex, ethnicity, education, income, employment, alcohol consumption, physical activity, current smoking, chronic kidney disease, and hypertension. Continuous values of blood glucose and blood pressure were also adjusted.


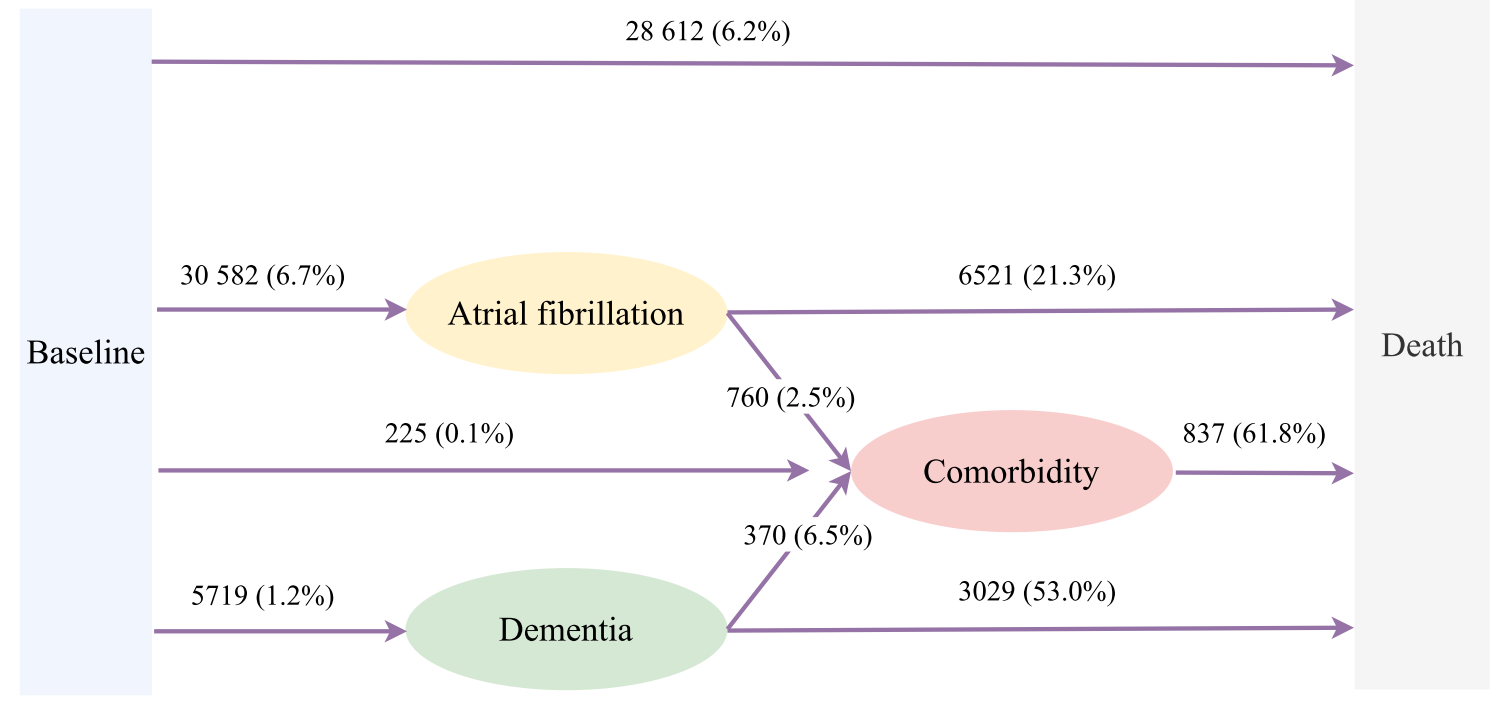


**Supplementary Figure 11.** Disease trajectories of atrial fibrillation and dementia, further including participants with same diagnosed dates of atrial fibrillation and dementia and therefore accounting for the transition from baseline to comorbidity.

Observed trajectories (expressed as numbers and percentages of participants in the previous stage) of atrial fibrillation and dementia.


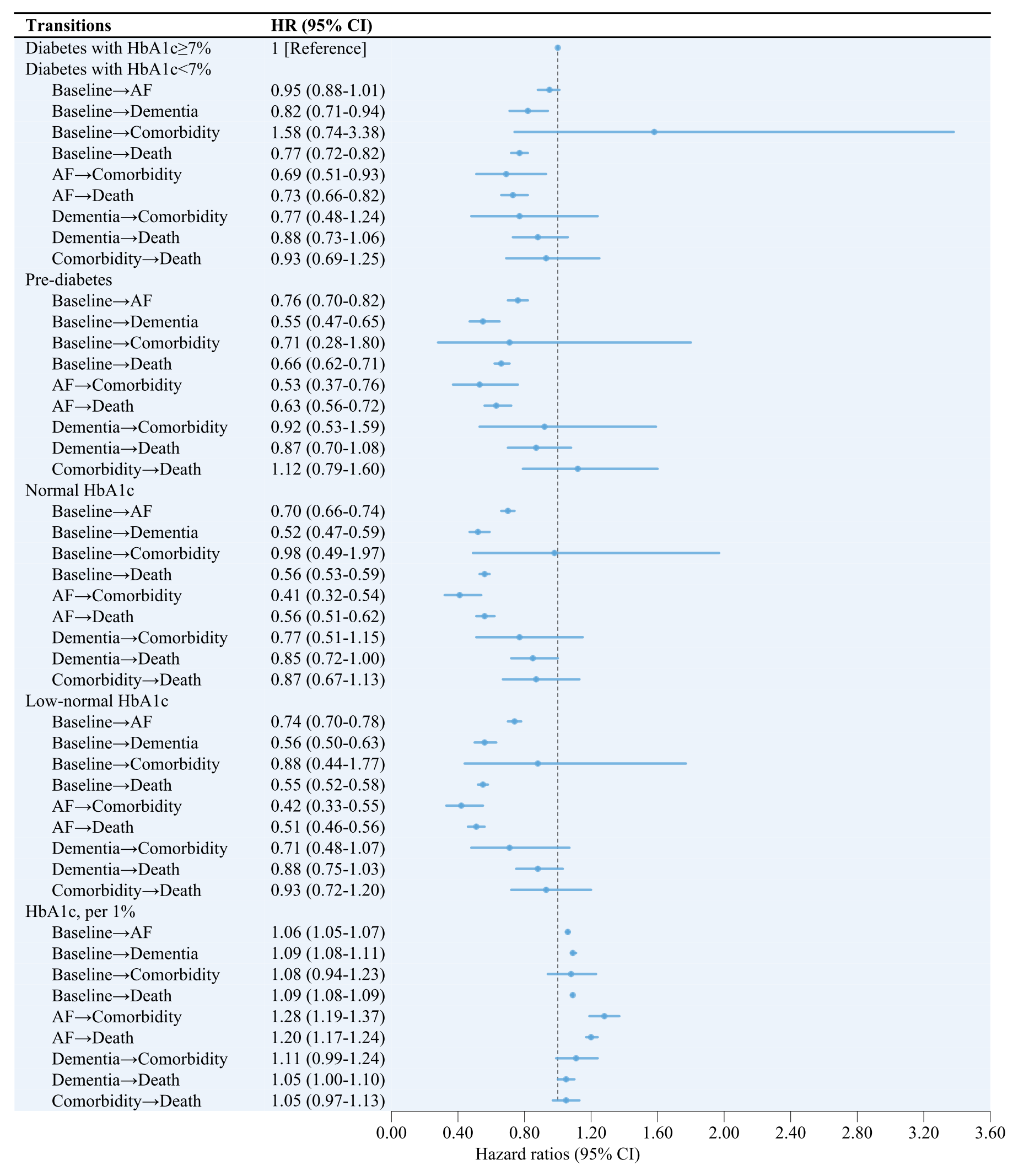


**Supplementary Figure 12.** Associations between glycemic status and disease trajectories of atrial fibrillation and dementia, further including participants with same diagnosed dates of atrial fibrillation and dementia and therefore accounting for the transition from baseline to comorbidity.

HbA1c, glycated hemoglobin; AF, atrial fibrillation; HR, hazard ratio; CI, confidence interval.

Hazard ratios of associations between glycemic status with different trajectories of atrial fibrillation and dementia, controlling for sex, ethnicity, education, income, employment, alcohol consumption, physical activity, current smoking, chronic kidney disease, and hypertension.
